# TCS: a web server for multiple sequence alignment evaluation and phylogenetic reconstruction

**DOI:** 10.1093/nar/gkv310

**Published:** 2015-04-08

**Authors:** Jia-Ming Chang, Paolo Di Tommaso, Vincent Lefort, Olivier Gascuel, Cedric Notredame

**Affiliations:** 1 Comparative Bioinformatics, Bioinformatics and Genomics Program, Centre for Genomic Regulation (CRG), Dr Aiguader 88, 08003 Barcelona, Spain; 2 Universitat Pompeu Fabra (UPF), 08003 Barcelona, Spain; 3 Institute of Human Genetics (IGH), UPR 1142, CNRS, 34396 Montpellier, France; 4 Institut de Biologie Computationnelle, LIRMM, UMR 5506: CNRS & Université de Montpellier, France

## Abstract

This article introduces the Transitive Consistency Score (TCS) web server; a service making it possible to estimate the local reliability of protein multiple sequence alignments (MSAs) using the TCS index. The evaluation can be used to identify the aligned positions most likely to contain structurally analogous residues and also most likely to support an accurate phylogenetic reconstruction. The TCS scoring scheme has been shown to be accurate predictor of structural alignment correctness among commonly used methods. It has also been shown to outperform common filtering schemes like Gblocks or trimAl when doing MSA post-processing prior to phylogenetic tree reconstruction. The web server is available from http://tcoffee.crg.cat/tcs.

## INTRODUCTION

The Transitive/Tree-based Consistency Score (TCS) web server allows biologists to estimate the local reliability of protein multiple sequence alignments (MSAs) for structural and evolutionary modeling purposes using the TCS metrics (1). The server makes it possible to colorize a pre-computed MSA according to its reliability and to filter out or down-weight unreliable positions for further evolutionary and structural analyses. A pre-computed MSA can be estimated from any available third party method. The web server is fully connected with the PhyML (2) tree reconstruction web server. In theory, it can also be used for DNA and RNA analyses though the method has only been validated on proteins so far.

The TCS server addresses an important need in the biological community. While the development of novel MSA methods has received a large amount of attention over the last two decades, the problem of quantifying the relative reliability of such alignments has been a less popular investigation topic. This is certainly surprising, given the approximate nature of all available algorithms and their frequently reported incapacity to deliver structurally correct models, even when using the best available methods (3). In practice, most publications describing novel MSAs come along with benchmarks that report the average accuracy of given software on specific benchmarks—usually made of structure-based MSAs. Given the variability in performance of even the best packages, these average figures are of little practical use for biologists. They merely allow bet hedging when picking up a given method, but they give no guarantee of estimating the best possible model on a given data set (4). This falls short of biologists’ expectations, which mostly involve assembling the best possible MSA or identifying the trust worthiest columns when doing homology or phylogenetic modeling.

The issue of identifying the most evolutionary or structural correct positions in an MSA has been addressed from two complementary perspectives. When doing phylogenetic reconstruction, the most widely used methods for MSA post-processing, Gblocks (5) and trimAl (6), filter out positions on the basis of their conservation and indel indexes. This filtering is done under the assumption that such positions are more likely to be inaccurately aligned. These methods have initially been validated on simulated data sets for their capacity to improve phylogenetic reconstruction although effectiveness of this approach was recently questioned in two independent reports (1,7). The most recent generation of methods (HoT (8), GUIDANCE (9), PSAR (10)) estimate reliability indexes by considering the stability of the MSA under some sort of re-alignment perturbation (sequence flipping, guide tree variation or sequence removal). TCS belongs to this group of methods and makes it possible to estimate the reliability of any position in an MSA by considering the stability of its alignment when independently re-aligning all possible pairs of sequences within the considered MSA.

In a recent publication (1), the TCS was extensively benchmarked for its capacity to identify positions in which the aligned residues are most likely to be structurally homologous. The results indicate that given the most widely used MSA methods, TCS is significantly more discriminative than HoT and GUIDANCE while being significantly faster than GUIDANCE. Regardless of the MSA methods, TCS can identify ∼40% of the correctly aligned residues with a confidence of 90% or higher. Using appropriate simulated and empirical benchmarks, we were also able to show that the proper use of the scoring positions—through up-weighting or filtering—makes it possible to build significantly more accurate phylogenetic trees, with most alignment methods reaching comparable accuracy levels when doing TCS-based post-processing. Altogether, these results indicate that the TCS reliability metrics can be used to improve both the structural and the evolutionary modeling capacity of most multiple sequence aligners. Its systematic usage should help decrease the uncertainty resulting from the use of different alignment methods and therefore increase the robustness and reproducibility of biological results established on the basis of MSA modeling.

## ALGORITHM

Given a pre-computed MSA, the TCS algorithm estimates the reliability of every pair of aligned residues (*PairTCS*), every individual residue (*ResidueTCS*), every MSA column (*ColumnTCS*), every MSA sequence (*SequenceTCS*) and the whole alignment (*AlignmentTCS*). The procedure implemented in the web server can be summarized as follows.

The first computation step involves extracting the sequences two by two in order to build a T-Coffee library. A T-Coffee library is a list of residue pairs weighted according to their probability to be part of the final MSA. These pairs do not have to be consistent with each other and a valid library can contain incompatible pairs. In theory users can compile these libraries using any suitable combination of pairwise and MSA methods. By default, the library is computed by applying a pair hidden Markov model adapted from ProbCons (11) onto every pair of sequences and by keeping all residue pairs having a posterior alignment probability higher than 0.9. For every pair of sequences, this roughly amounts to select the optimal alignment along with a few high scoring sub-optimal alignments. Other library schemes are supported including Fast-Coffee that involves running three fast aligners onto the sequences (MAFFT (12), MUSCLE (13) and Kalign (14)) and populating the library with all pairs of residues aligned in the three resulting MSAs. The library computation is the most computationally intense step and therefore the most limiting. The running time of default library requires *O*(*N*^2^*L*^2^), where *N* is the number of sequences and *L* is the length of the MSA.

The library is then used to estimate the TCS score of every pair of aligned residues in the target MSA using the following procedure (see (1) for details). Given two aligned residues *R^x^_i_* (*i*th residue of sequence *x*) and *R^y^_j_* (*j*th residue of sequence *y*) forming the (*R^x^_i_*,*R^y^_j_*) pair within the target MSA, the TCS score is estimated by identifying within the library all compatible pairs, *R^x^_i_R^z^_k_* and *R^z^_k_R^y^_j_*, containing a common intermediate residue, *R^z^_k_*, from a third sequence *z*. Two compatible pairs define a triplet whose score is assigned the lowest score of the two pairs. All triplets’ scores are then added up and the resulting sum is normalized by the sum of the scores of all the pairs involving either *R^x^_i_* or *R^y^_j_* ; thus yielding a value between 0 and 1 named *PairTCS* of (*R^x^_i_*,*R^y^_j_*).

The other metrics are directly derived for the *PairTCS*. The *ResidueTCS* score is assigned to every residue and obtained by averaging the *PairTCS* score over all pairs involving the considered residue and any another residue appearing in the same column of the target MSA. The *ColumnTCS* score is estimated by averaging the *PairTCS* score over all pairs of aligned residues in each target MSA column. The *SequenceTCS* and the *AlignmentTCS* are obtained by averaging all the *ResidueTCS* in the sequence and over the entire alignment, respectively. The *ResidueTCS, ColumnTCS, SequenceTCS* and *AlignmentTCS* are available in *score_ascii* output format of the web server. The *PairTCS* is not a default output (*sp_ascii*) and is only available using the command line version of T-Coffee (http://tcoffee.readthedocs.org/en/latest/TCS.html).

Once the metrics have been computed, they are used for filtering out the less reliable columns (or residues), coloring every position in the target MSA and generating bootstrap replicates (for tree reconstruction) using the *ColumnTCS* as a weight when randomly selecting columns for inclusion in a bootstrap replicate with the same column size of the target MSA (1). The filtered MSAs and the bootstrap replicates can be either downloaded for further usage or sent to the PhyML web server. PhyML can accept the replicates input as multiple data sets (option ‘Number of data sets’ in PhyML server) and it will generate an individual tree for each replicate instead of the consensus-bootstrap tree.

### TCS WEB SERVER

The TCS web server is part of the T-Coffee web platform; its access is free and unrestricted, without login procedure. The server is accessible from http://tcoffee.crg.cat/tcs with any standard web browser (Mozilla Firefox 5+, Google Chrome, Internet Explorer 8+, Safari 6+ and Opera 11+). All the functions of the server are also available through the command line version of T-Coffee, freeware open-source software available from http://www.tcoffee.org/Projects/tcs/. Detailed usage information can be found on the same page or http://tcoffee.readthedocs.org/en/latest/TCS.html.

### Input

**Figure 1.**
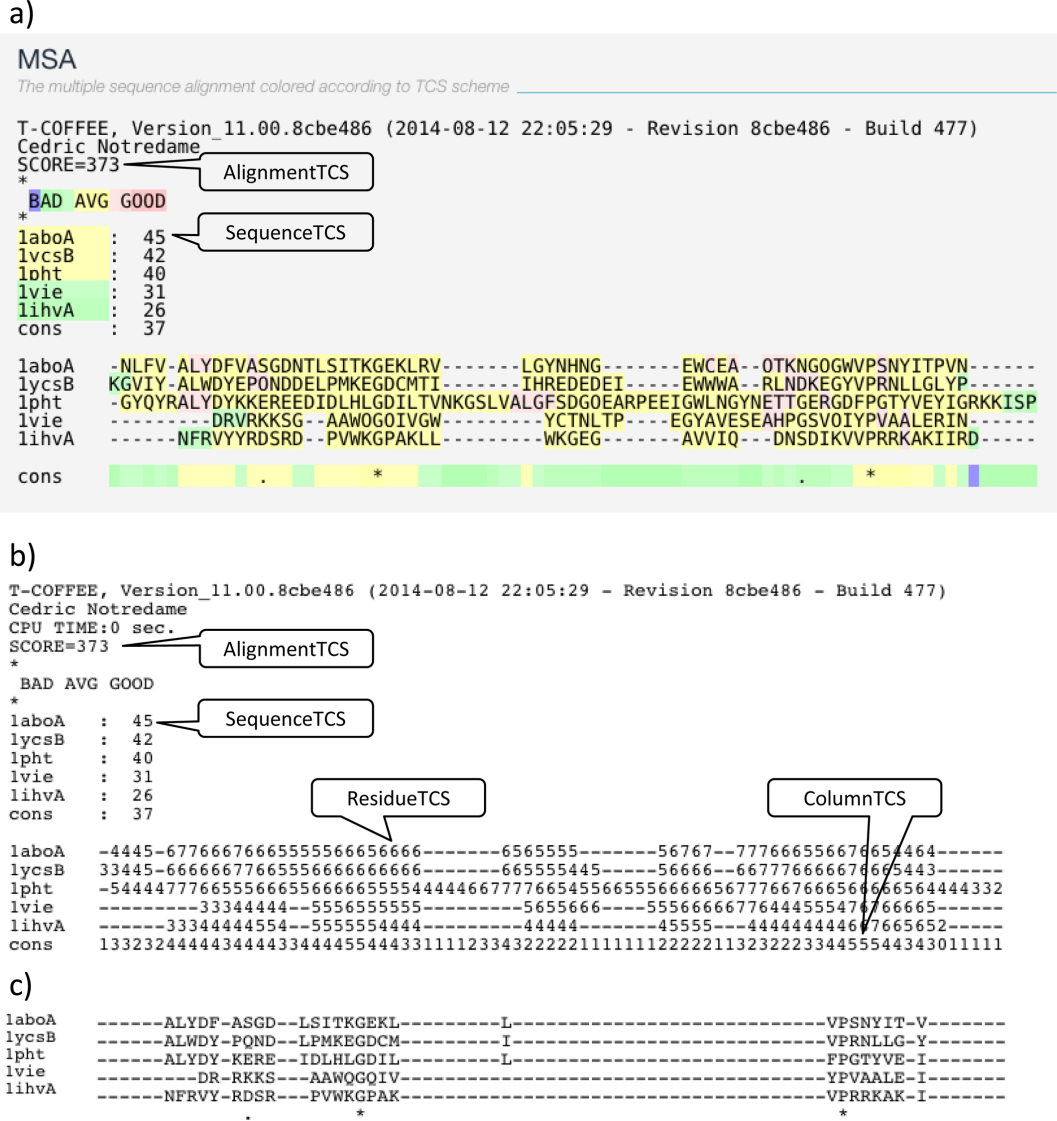
(**a)** Graphic output (*score_html*). The SCORE is the *AlignmentTCS* normalized to a maximum of 1000. The sequence list below indicates the relative *SequenceTCS* of the considered sequences with values normalized to a maximum of 100. In the alignment below, residues are color coded from blue (*ResidueTCS* = 0) to dark pink (*ResidueTCS* >= 9). The line below the alignment indicates the consensus score of every column (*ColumnTCS*). **(b)***score_ascii* output of the same alignment. Residues are replaced by their *ResidueTCS* normalized between 0 and 9. **(c)** Filtered output in CLUSTAL format. Columns having *ColumnTCS* less than 4 have been removed (default). The advanced option, Remove empty columns, is disable such that empty columns are kept for illustration purposes.

MSAs (CLUSTAL, FASTA, MSF, Phylip or Nexus format) can either be cut/pasted or uploaded. For unaligned sequences, users can run any T-Coffee method first and then click a blue button ‘Core/TCS’ in the ‘Send results’ section, which will automatically initialize pre-filled TCS service with T-Coffee MSA as input. By default the server is set to compute a *Mproba_pair* T-Coffee pairwise library and to generate the filtered alignments along with its other outputs by removing all columns having a scaled *ColumnTCS* score (range 0–9) less than 4. Users can change this behavior by triggering the ‘Show more options’ hyperlink that makes it possible to change the library computation methods, the filtering threshold and/or the filtering on residues rather than columns. The advance mode also makes it possible to retain empty columns after filtering (Figure 1c). Email address is optional but advisable when submitting computationally intensive jobs. The ‘Submit’ button triggers the job processing.

### Computation

The main limiting step of TCS is the library computation, which involves computing all possible pairwise alignments on the input sequences. On a MSA of seven sequences, the server takes ∼20 s—most of it being queuing access. It can deal with a MSA of up to 1000 sequences though users are encouraged to select fast library computation modes through the ‘Show more options’ link (i.e. *Mkalign_msa*, *Mmafft_msa* and *Mmuscle_msa*) when processing MSAs containing more than 100 sequences. This mode is significantly faster. It is used by Ensembl compara (15) and takes ∼3 h to process a MSA containing 1000 sequences.

The browser does not need to stay open. Thanks to a cookie system private to the user's computer, the ‘History’ link on the top bar provides access to earlier computation results whenever the TCS page is re-opened. User can also retrieve their results using the permanent URL http://tcoffee.crg.cat/apps/tcoffee/result?rid=jobid, where *jobid* is the ID given at the time of submission. Results are kept on the web server up to one month after computation.

### Output

The server final output is a summary page containing all the result files, the parameters used for running and the downstream phylogenetic reconstruction. The summary displays the following sections in order:

- Citation: list of articles related to TCS.
- MSA: a colorized version of the MSA reliability (Figure 1a. *score_html*).
- Result files: a series of output files that include: *score_html* the html format and *score_ascii* its plain text format indicating the *ResidueTCS, ColumnTCS, SequenceTCS* and *AlignmentTCS* scores, respectively (Figure 1b. *score_ascii*). The ‘Multiple Alignment’ subsection provides a filtered version of the original MSAs in CLUSTAL, FASTA and Phylip formats (Figure 1c. filtered MSA) that can be used for phylogenetic reconstruction or homology modeling. This section also provides two files: named *tcs_weighted* in which all columns are duplicated a number of times equal to their *ColumnTCS*; and the *tcs_replicate* that contains a 100 bootstrap replicates of the original MSA with column randomly selected weighed by their *ColumnTCS*. These two files can be also used for the phylogenetic reconstruction. All files can be downloaded as a single zip file or copied to the user's Dropbox account.
- Phylogenetic reconstruction: this section makes it possible to send the filtered MSA, the weighted MSA and the bootstrap replicates to the PhyML web server. The buttons will trigger the automatic upload of a pre-filled PhyML form, leaving the user to either launch the job or modify the default parameters.
- Send results: forward these results to other online tools.
- Info: some information related to the submitted job including running time and date.
- Replay: re-run the job while modifying input options or data. It also provides command line option for users to utilize the full featured TCS.
- Feedback: share user's experience on the social networks.

## DISCUSSION

We describe the TCS server, a web-based tool able to identify the most correct regions within any pre-computed MSA, regardless of the chosen aligner. To the best of our knowledge, TCS is the first reliability index that has been validated for its relevance at improving both homology modeling and phylogenetic reconstruction. The TCS server also makes it possible to depart from using the standard similarity based filtering schemes of Gblocks or trimAl thus allowing more information to be retained when estimating trees. Overall, this server is able to reduce alignment error (uncertainty) on downstream biological analyses. Future improvements will extend TCS capacity to handle RNA alignment.
